# Forensic age estimation in living children: how accurate is the Greulich-Pyle method in Sabah, East Malaysia?

**DOI:** 10.3389/fped.2023.1137960

**Published:** 2023-06-15

**Authors:** Khin Mya Nang, Abdul Jabbar Ismail, Anithaa Tangaperumal, Aye Aye Wynn, Tin Tin Thein, Firdaus Hayati, Yong Guang Teh

**Affiliations:** ^1^ Department of Pathology & Microbiology, Faculty of Medicine & Health Sciences, Universiti Malaysia Sabah, Kota Kinabalu, Malaysia; ^2^ Department of Anaesthesiology, Faculty of Medicine & Health Sciences, Universiti Malaysia Sabah, Kota Kinabalu, Malaysia; ^3^ Department of Radiology, Serdang Hospital, Kajang, Malaysia; ^4^ Department of Surgery, Faculty of Medicine & Health Sciences, Universiti Malaysia Sabah, Kota Kinabalu, Malaysia; ^5^ Department of Radiology, Faculty of Medicine & Health Sciences, Universiti Malaysia Sabah, Kota Kinabalu, Malaysia

**Keywords:** bone age, forensic age, Greulich-Pyle atlas, Sabah, radiograph

## Abstract

**Background:**

The Greulich and Pyle's Radiographic Atlas of Skeletal Development of the Hand and Wrist (GP Atlas) is the most widely used method of determining the bone age (BA) of a child. It is also a widely accepted method for forensic age determination. As there is limited local bone age data for forensic age estimation, the purpose of this study was to assess the accuracy of the GP Atlas for forensic age determination in living Sabahan children.

**Method:**

This study recruited 182 children between the ages of 9 years to 18 years. BA estimation of the left-hand anteroposterior radiographs were performed by two experienced radiologists using the Greulich-Pyle method.

**Results:**

The BA estimates from two radiologists had very high interobserver reliability (ICC 0.937) and a strong positive interobserver correlation (r > 0.90). The GP method, significantly and consistently underestimated chronological age (CA) by 0.7, 0.6 and 0.7 years in overall children, boys and girls respectively with minimal errors. Mean absolute error and root of mean squared error for overall children was 1.5 and 2.2 years respectively, while mean absolute percentage error was 11.6%. This underestimation was consistent across all age groups but was statistically significant only at 13–13.9 and 17–18.9 years old age groups.

**Conclusion:**

Despite high interobserver reliability of BA estimation using the GP Atlas, this method consistently underestimates the age of the child in all children to a significant degree, for both boys and girls across all age groups, with an acceptably low level of error metrics. Our findings suggest that locally validated GP Atlas or other type of assessments (artificial intelligence or machine learning) are needed for assessment of BA to accurately predict CA, since current GP Atlas standards significantly underestimated chronological age with minimal error for children in Sabah. A larger population-based study would be necessary for establishing a validated atlas of a bone age in Malaysia.

## Introduction

Greulich and Pyle's Radiographic Atlas of Skeletal Development of the Hand and Wrist (GP Atlas) is the most widely used method of determining the bone age (BA) of a child (1). This system relies on radiographic findings associated with developmental maturity that are presented in a series of standard radiographs of the left hand according to gender. The documented accuracy of the GP Atlas is between 0.6 year and 1.1 years in Caucasian children.

The GP Atlas is also a widely accepted method for forensic age determination (2). Experts agree that this method is reliable and easily accessible, as it only utilizes a single anteroposterior radiograph of the left hand (3). However, many studies have demonstrated that the GP Atlas overestimates the chronological age (CA) of non-Caucasian children (2, 4). Deviation of as much as 1.1 years has been reported in Turkish females older than 15 years of age (5). Apart from ethnicity, nutritional status has also been shown to influence the accuracy of the GP Atlas, particularly in resource-limited developing countries (6). Nevertheless, skilled interpretation of the GP Atlas can be very useful especially when locally validated standards are used (7).

The United Nations High Commissioner for Refugees (UNHCR) defines a child as an individual who is younger than 18 years of age (8). As children are given special privileges in the UNHCR charter, forensic determination of childhood status carries important legal ramifications, particularly for refugees without appropriate documentation. Other possible medico-legal applications of the GP Atlas method include providing independent age estimates for criminal trials and immigration purposes (9). However, despite its apparent usefulness, there is limited data on the accuracy of the GP Atlas in Southeast Asian countries, and particularly Sabah in East Malaysia.

As such, the purpose of this study was to assess the accuracy of the GP Atlas method for forensic age determination in living Sabahan children.

## Methods

This study complies with standards set by the Medical Research Ethics Committee, Universiti Malaysia Sabah [JKEtika 1/20(24)]. Data was collected from the UMS community clinic, an established healthcare facility under the UMS Teaching Hospital system. All images were acquired between August 2021 and August 2022. Written informed consent was obtained from the parents of the children who participated in this study.

### Patients enrolment

Patients between the ages of 9 to 18 years old were chosen for two reasons: (1) because forensic age determination is a challenge in this age group and (2) we regularly see children of this age group in our community clinic. The inclusion criteria for this study were (1) children between the ages of 9 and 18 years of age, (2) no history of congenital abnormalities or other medical illness, (3) no history of fracture to the left hand, (4) no soft tissue abnormalities and (5) normal clinical findings in both hands. BA estimates were made using the GP Atlas method by two experienced radiologists working at a tertiary children's hospital.

### Image acquisition and bone age assessments

Images were acquired using a Toshiba KXO-32R general radiography machine. Radiographs of the left hand were acquired using the following exposure settings: (1) 46–50 kVp, (2) 2.0 mAs, (3) SID 100 cm and (4) with no grid. Images were read on a vendor approved console. Independent BA estimations by two radiologists with 4 years of clinical experience in pediatric radiology. BA estimates were obtained using the Greulich and Pyle's Radiographic Atlas of Skeletal Development of the Hand and Wrist (1). Both radiologists were blinded to the CA of the patients.

### Data analysis

Data analysis was performed on SPSS version 28. Data from this study were mainly continuous numerical data, thus the normally distributed data is presented as mean and standard deviation (SD) and skewed data presented as median and interquartile range (IQR).

Descriptive statistics obtained from the data, such as actual CA and BA, were presented as mean or median and standard deviation (SD) or interquartile range (IQR) depending on the normality of data distribution. Participants were categorized into gender groups (boys and girls) and age groupings of 9–10, 10–11, 12–13, 14–15, 16–17, and 18–19 years.

Interobserver reliability of BA measurements was assessed using intraclass correlation coefficient (ICC), with two-way fixed effect model, single measures, and absolute agreement settings in SPSS (10). Value of >0.90 was considered an adequate measure of precision to ensure reliability of the assessment by the two radiologists, and has been used in previous studies conducted for bone age assessments using Greulich-Pyle methods (10–12). Pearson correlation analysis was performed between the BA values from two radiologists to ascertain the correlation strength between the two readers. Other correlation analyses were also performed between BA and CA of all children, and also specifically in boys and girls. Interpretation of the Pearson correlation coefficient (r) followed statistical standards as described by Patrick Shober and colleagues (13). Pearson correlation coefficient showed strong correlation between the BA values from two radiologists, and a mean BA value for each child was computed using SPSS by dividing the sum of the BAs by 2.

Analysis of accuracy between CA and mean BA was performed for the entire cohort using paired sample t-test to determine any statistical significant differences between CA and BA. Subgroup analyses were also performed for boys, girls, and different age groups. This method has been used in previous studies in other specific pediatric populations in Turkey and India (4–6).

Further analysis of accuracy was performed by measuring the mean absolute difference between CA and BA in order to improve the calculation method used previously in studies in Turkish, Indian and Iranian children (5–7). Mean absolute error (MAE), root of mean squared error (RMSE) and mean absolute percentage error (MAPE) were also calculated as part of the error metrics.

In the subgroup analysis of age groups, a non-parametric test was performed using the Wilcoxon Matched-Pair Test in SPSS to detect any significant differences between CA and BA. Parametric tests were not used because the number in each group was less than 30 individuals. This analysis was repeated for each age subgroup. In each age subgroup, we could not perform further analysis for boys and girls because of insufficient sample size.

Multiple linear regression was performed to derive a formula for predicting forensic CA for local Sabahan children.

## Results

The study enrolled 182 children between the ages of 9 to 18 years. Table 1 shows the distribution of the 182 children enrolled in this study, of which 103 were girls and 79 were boys. The mean chronological age of overall participants was 13.9 ± 2.4 years old. The mean chronological age for girls was 13.6 ± 2.3 and boys were 14.2 ± 2.6 years old.

**Table 1:** Main study findings of bone age and chronological age differences with measurement of error metrics of accuracy.

Gender	Frequency (%)	CA, mean ± SD	BA mean ± SD	CA–BA mean Difference ± SD	Paired *t*-test	Cohen's d	MAE	RMSE	MAPE
Overall	182 (100%)	13.9 ± 2.4	13.2 ± 3.1	0.7 ± 2.0	t 4.7, r 0.76, *p* < 0.01	1.98	1.5	2.2	11.6%
Boys	79 (43.4)	14.2 ± 2.6	13.6 ± 3.0	0.6 ± 1.7	t 3.1, r 0.81, *p* < 0.01	1.76	1.4	1.9	10.6%
Girls	103 (56.5)	13.6 ± 2.3	12.9 ± 3.1	0.7 ± 2.2	t 3.5, r 0.72, *p* < 0.01	2.15	1.6	2.3	12.3%

All units are in years, to 1 decimal point, ± indicates standard deviation.

MAE, mean absolute error; RMSE, root of mean squared error; MAPE, mean absolute percentage error.

**Figure 1:**
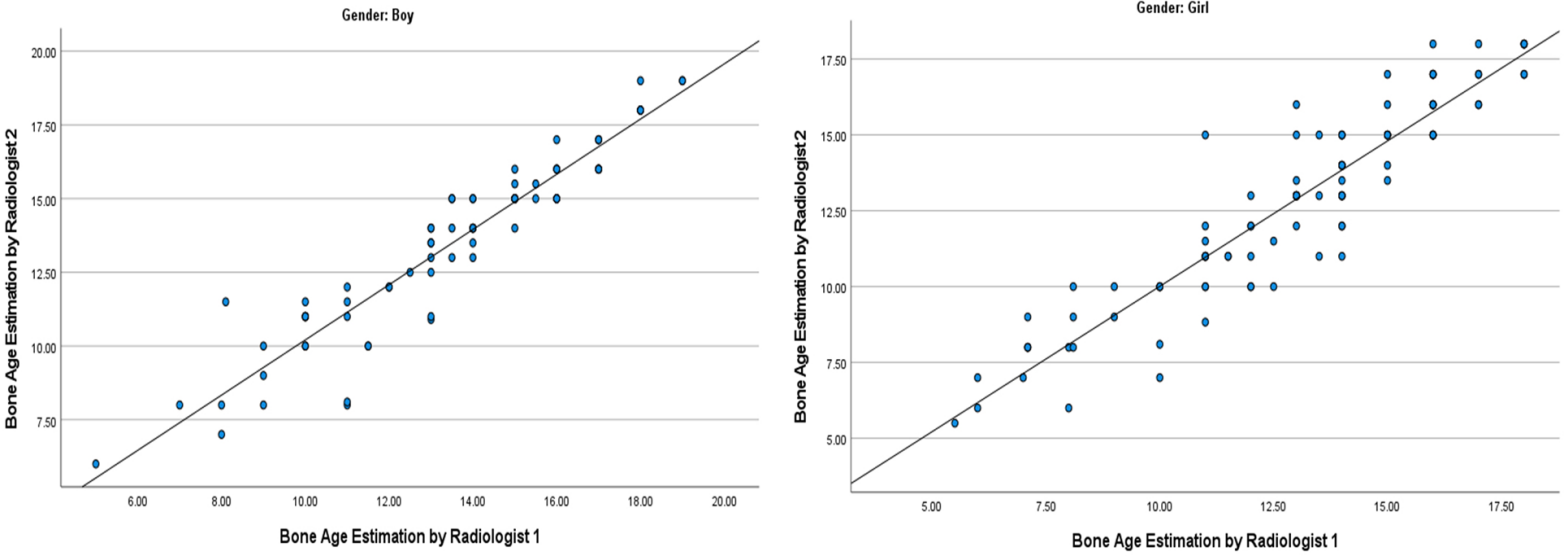
Graphs showing high interobserver agreement when estimating bone age of boys and girl. The interobserver bone age assessments were strongly correlated for boys, r(75) = .901, *p *< 0.001, and for girls, r (100) = .782, *p *< 0.001.

Assessment of precision showed excellent inter-rater reliability between the two radiologists for all children, as well as in the subgroup analysis of boys and girls. ICC for the two radiologists was 0.937, indicating excellent inter-rater reliability with an F ratio of 0.804 (*p* = 0.37). The ICC was also high for boys (ICC 0.947, F 0.03, p 0.86) and also girls (ICC 0.93, F 0.96, *p* 0.33). In Figure 1, the interobserver bone age assessments were very strongly correlated for boys, r(79) = 0.946, *p *< 0.001, and for girls, r (103) = 0.931, *p *< 0.001.

**Figure 2:**
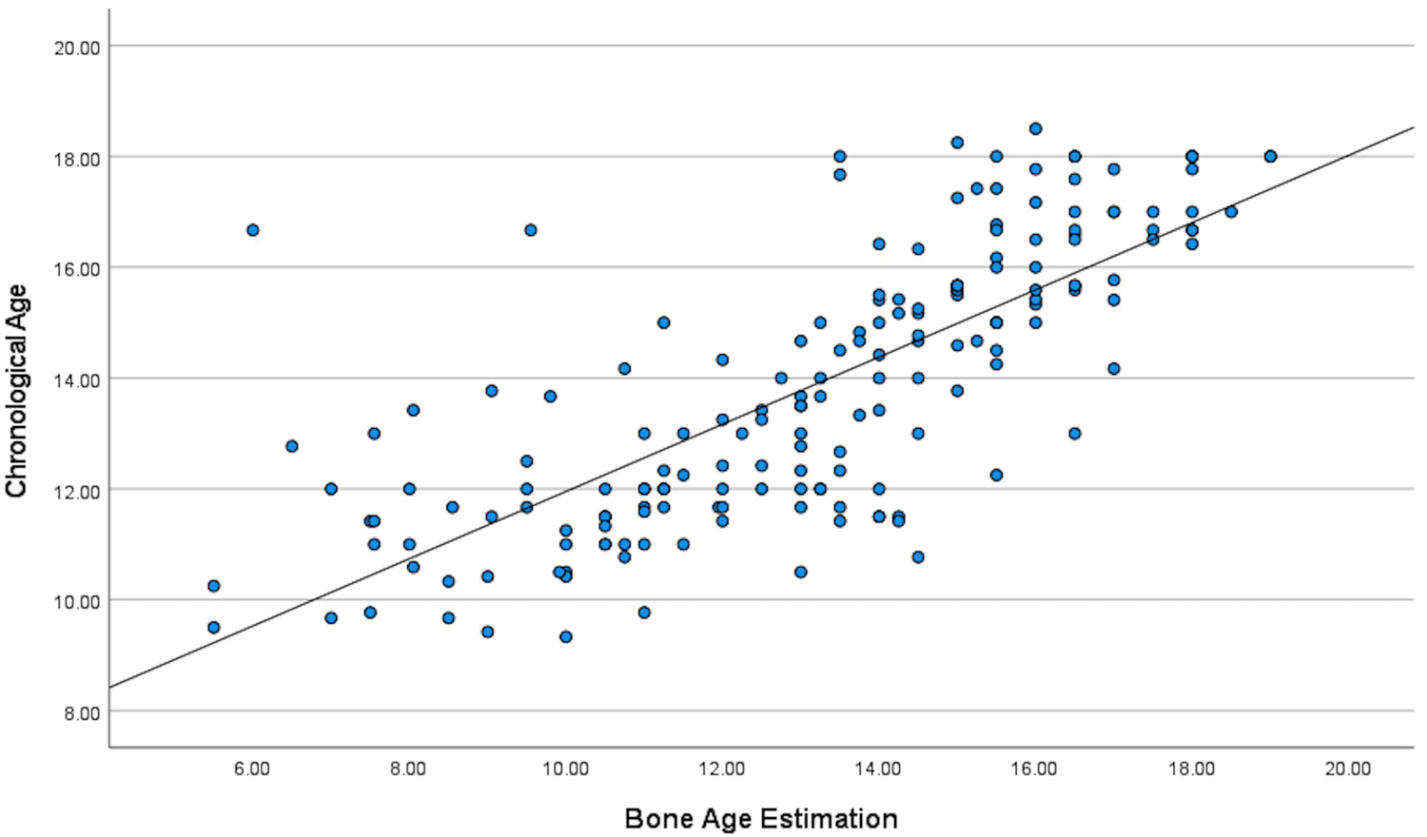
Pearson correlation study indicating statistically significant positive correlation between bone age estimation using GP method and actual chronological age.

Strong correlation between BA estimation with actual CA can be seen when the entire cohort was included, r(182) = 0.763, *p* < 0.001 (Figure 2). When boys & girls were analyzed separately, both showed strong correlation between BA and CA, but Pearson correlation coefficient (r) value was higher in boys [r(79) = 0.81, *p* < 0.01], indicating stronger correlation in boys compared to girls [r(103) = 0.723, *p* < 0.01 for girls].

There were statistically significant differences between BA estimates and CA in general, as well as for boys and girls separately. BA underestimated CA by 0.7 years for all children in general. In term of gender difference, BA underestimation of CA was smaller in boys as compared to girls (0.6 vs. 0.7 years). Standard deviation for all 3 assessments above in terms of BA and CA differences were between 1.7 to 2.0 years indicating high variance in differences between BA and CA. Since the difference between BA and CA was statistically significant, further assessment of accuracy was conducted by measuring errors of BA assessment using the GP method to predict the chronological age of the children. MAE, RMSE and MAPE for overall children were 1.5 years, 2.2 years and 11.6% respectively; for boys it was 1.4 years, 1.9 years and 10.6%; and for girls 1.6 years, 2.3 years and 12.3% respectively.

Further subgroup analysis was done for all age subgroups. Table 2 shows the number of cases for each age group, where the 11.0–11.9 years old group had the highest number of cases, 30 cases, while the rest of the groups were less than 30 with 9.0–9.9 years old the lowest number of cases at 7 cases.

**Table 2:** Accuracy assessment of GP method for bone age assessment to predict chronological age.

Age groups	Cases	CA median (IQR)	BA median (IQR)	BA–CA difference, median	Wilcoxon matched pairs test	AE median (IQR)	APE median (IQR)
9.0–9.9	7	9.7 (9.4–9.7)	8.5 (7.0–8.5)	−1.2	Z −1.35, p 0.176	1.5 (0.67–2.67)	15.5% (7.1–27)
10.0–10.9	10	10.5 (10.4–10.6)	10.0 (8.4–11.3)	−0.5	Z −1.17, p 0.241	1.6 (0.7–2.8)	15.6% (6.4–26)
11.0–11.9	30	11.5 (11–11.7)	10.9 (9.9–12.3)	−0.6	Z −1.24, p 0.214	1.2 (0.7–2.6)	10.1% (6.4–22)
12.0–12.9	25	12.0 (12–12.3)	12.0 (10.7–13.1)	0	Z −0.97, p 0.331	1.1 (0.8–2.3)	8.7% (6.2–19)
13.0–13.9	20	13.4 (13–13.6)	12.8 (11.1–13.6)	−0.6	Z −2.05, p 0.04	1.2 (0.5–3.1)	9.2% (3.8–24)
14.0–14.9	18	14.5 (14.1–14.7)	14.0 (13.2–15.1)	−0.5	Z −1.16, p 0.246	1.0 (0.5–1.4)	6.6% (3.4–10)
15.0–15.9	28	15.4 (15.0–15.7)	15.0 (14.3–16.0)	−0.4	Z −1.22, p 0.222	0.7 (0.6–1.1)	4.7% (4.6–7.3)
16.0–16.9	18	16.5 (16.4–16.7)	16.0 (15.3–17.5)	−0.5	Z −1.22, p 0.255	1.1 (0.5–1.6)	6.5% (3.0–10)
17.0–17.9	15	17.3 (17.0–17.7)	16.5 (15.5–17.5)	−0.8	Z −2.03, p 0.043	1.1 (0.5–1.9)	6.2% (3.0–11)
18.0–18.9	11	18.0 (18.0–18.0)	16.5 (15.5–18.0)	−1.5	Z −2.11, p 0.035	1.5 (0.0–2.5)	8.3% (0–14%)

All units are in years to 1 decimal point except last column on the right,.

AE, absolute error; APE, absolute percentage error.

There were statistically significant differences between BA and CA in the age groups of 13.0–13.9 years (−0.6 years), 17.0–17.9 (−0.8 years), and 18.0–18.9 years (−1.5 years). MAE and MAPE were 1.2 years (IQR 0.5–3.1) and 9.2% (3.8–24) respectively for the age group of 13.0–13.9 years old. For the age group of 17.0–17.9 years old, MAE and MAPE were 1.1 years (IQR 0.5–1.9) and 6.2% (3–11) respectively. For the age group of 18.0–18.9 years old, the MAE and MAPE were 1.5 years (IQR 0–2.5) and 8.3% (0–14) respectively.

**Figure 3:**
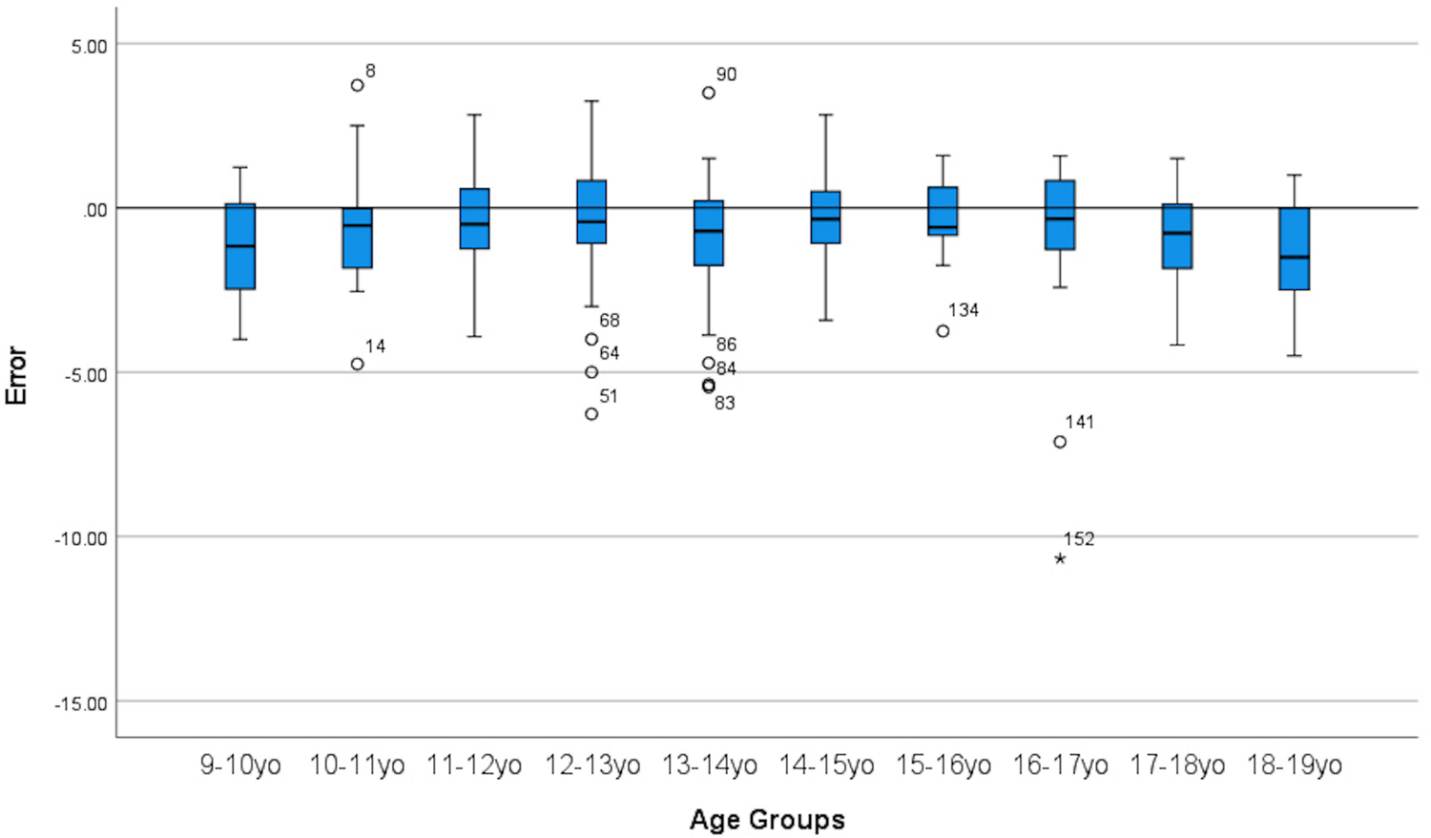
Boxplot showing GP method for bone age assessment consistently underestimate chronological age.

Figure 3 displays a boxplot demonstrating consistent underestimation of chronological age across all age groups. However, only age groups 13.0–13.9, 17.0–17.9 and 18.0–18.9 years had statistically significant underestimation.

**Figure 4:**
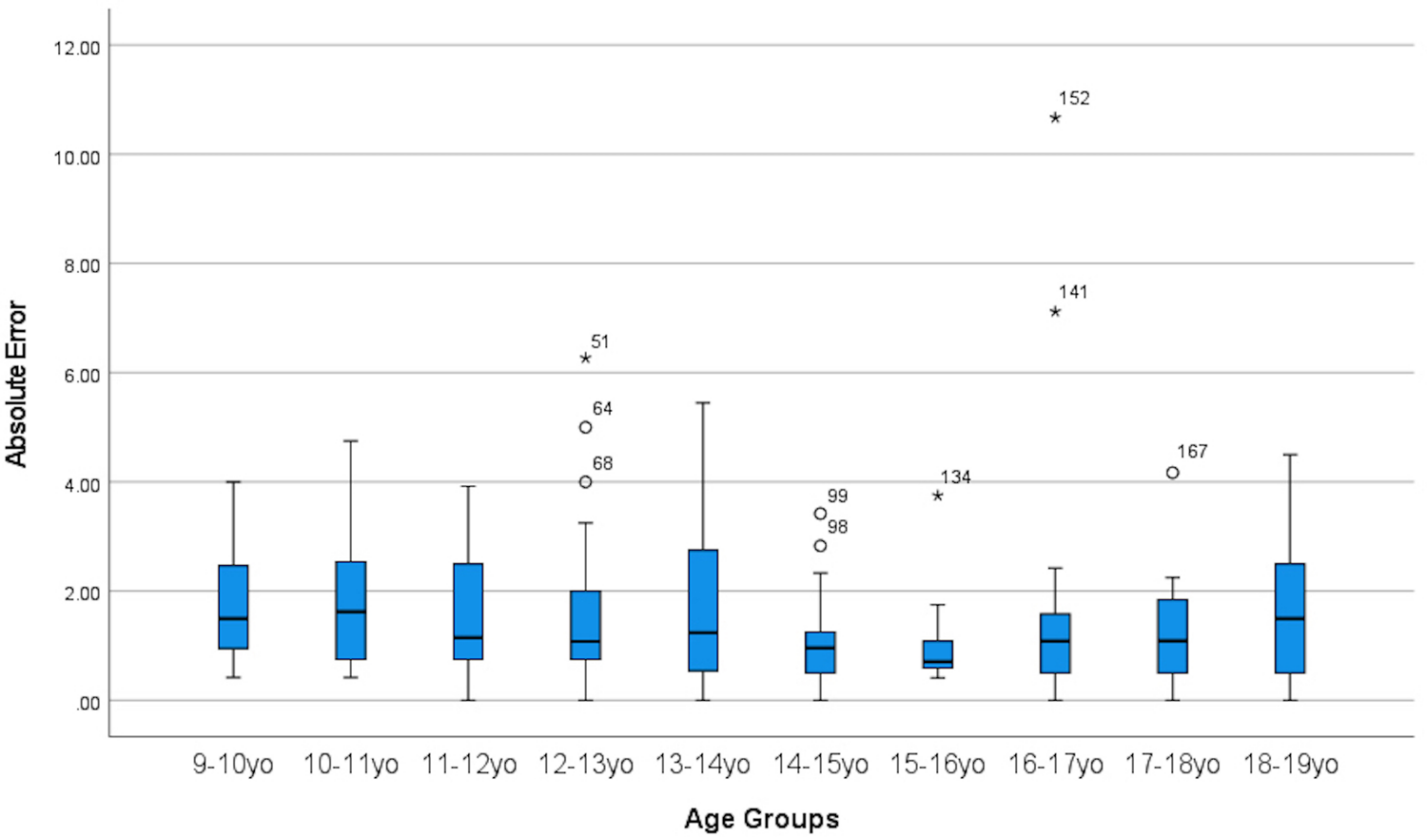
Boxplot showing absolute error between CA and BA across all age groups.

**Figure 5:**
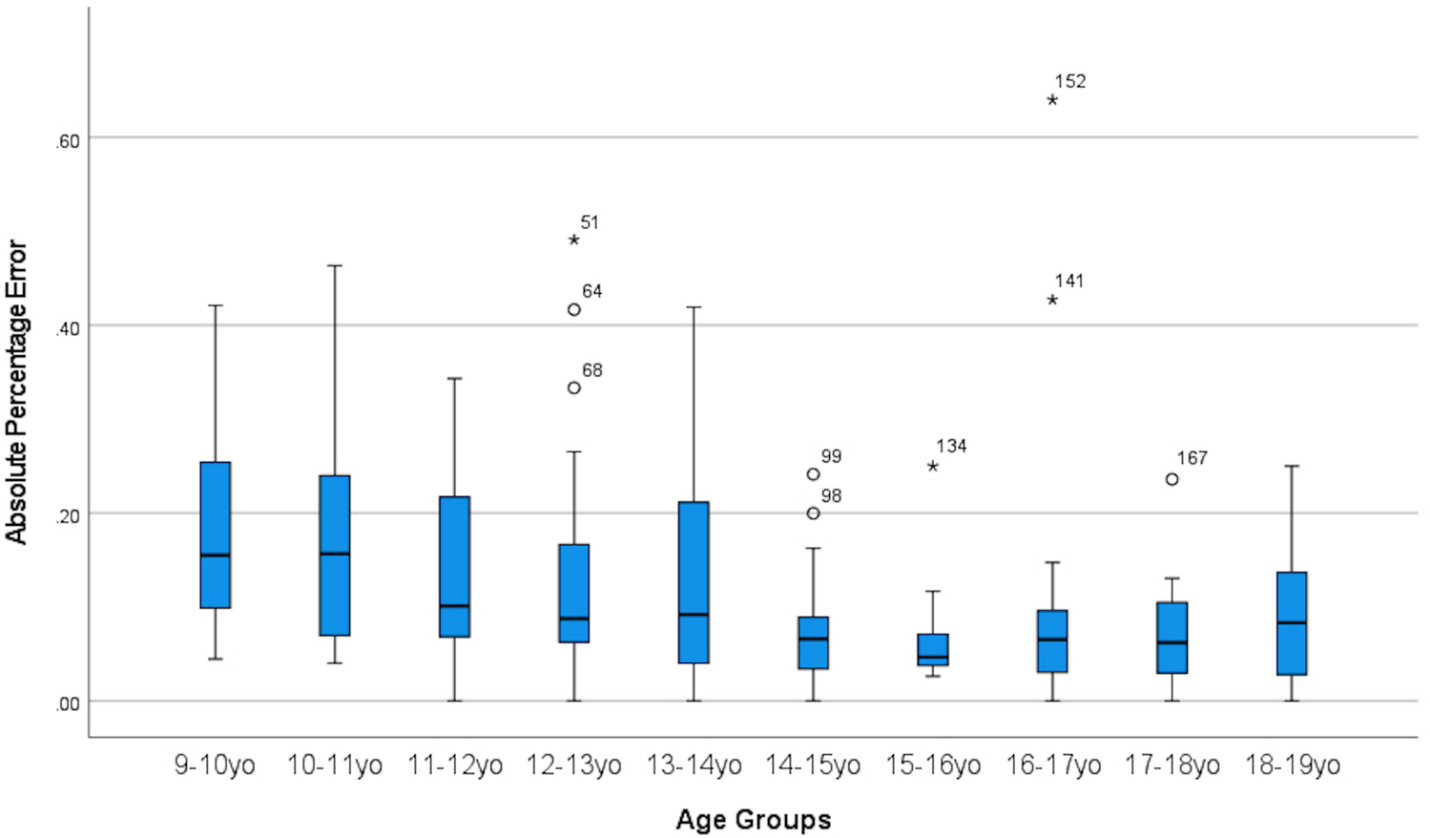
Boxplot showing absolute percentage error between CA and BA across all age group.

Figures 4 and 5 displays the metrics of absolute error and absolute percentage error for all age groups and it can be seen that there were high amounts of variance present as well as outliers.

Multiple linear regression demonstrated that gender was not a factor in predicting forensic CA, and was excluded. Subsequently, we proceeded with generation of predictor modeling using simple linear regression analysis as BA remained the only statistically significant predictor of CA in overall children. BA was deemed to be statistically significant and was able to predict chronological age with R-square of 0.582 and B coefficient of 0.607, *p* < 0.01 (95% CI 0.53–0.68). Tables 3 and 4 display the result of the multiple linear regression and simple linear regression respectively. The regression equation to improve prediction of actual chronological age of children is sabah using the GP method is CA = BA*0.607 + 5.889.

**Table 3:** Multiple linear regression to explore prediction of actual chronological age when using GP method for bone age assessment.

Independent variables	Beta coefficient	95% CI for beta coefficient		*p*-value	R Square
		LB	UB		
(Constant)	6.134	4.805	7.463	<0.01	0.583
Bone age	0.604	0.528	0.68	<0.01	
Gender	−0.137	−0.608	0.333	0.566	

**Table 4:** Simple linear regression for prediction of actual chronological age using GP method for bone age assessment.

Independent variables	Beta coefficient	95% CI for beta coefficient		*p*-value	R Square
		LB	UB		
(Constant)	5.889	4.862	6.915	<0.01	0.582
Bone age	0.197	0.531	0.682	<0.01	

**Table 5:** Simple linear regression for prediction of actual chronological age using GP method for bone age assessment when boys and girls treated separately.

Independent variables	Beta coefficient	95% CI for beta coefficient		*p*-value	R Square
**For Boys**		LB	UB		
(Constant)	4.819	3.24	6.398		0.656
Bone age	0.691	0.58	0.805	<0.01	
**For Girls**					
(Constant)	6.655	5.29	8.02	<0.01	0.522
Bone age	0.543	0.44	0.65	<0.01	

After performing simple linear regression for boys and girls separately, the results indicate that BA was able to predict chronological age with different regression equations. Regression equation to predict chronological age for children in Sabah were Forensic CA_boy_ = 4.819 + (0.691*BA) and Forensic CA_girl_ = 6.655 + (0.543*BA). Details on the simple linear regression for boys and girls presented in Table 5.

## Discussion

### Precision of GP method in bone age estimation in children in Sabah

Our data, in terms of the precision of the GP method to predict forensic CA, has excellent inter-rater reliability based on the high ICC value of >0.9 for overall measurements and for the subgroups of boys and girls. ICC Value of >0.9 in assessing reliability is considered excellent, based on established statistical convention (14). There was also strong inter-rater correlation in terms of BA assessments between the two radiologists using GP method for forensic CA determination, indicated by Pearson correlation coefficient of >0.9 for both boys and girls. The results of our study demonstrate that the GP method can reliably predict forensic CA in children similar to other methods of assessing bone age (12, 15). Our findings are in agreement with other studies that have investigated the use of the GP method to estimate forensic CA, which have ICC > 0.9 or strong Pearson correlation between assessments by two or more radiologists (4, 16, 17).

### Correlation between bone age and chronological age when using Greulich-Pyle method

Current evidence has been strong for the GP method when assessing BA to predict forensic CA in children. Our findings are consistent with other international studies—for example one study conducted in Türkey by Büken and colleagues obtained an r value of 0.882 for girls and 0.9 for boys (5). Relatedly, another Korean study has also shown a strong correlation between BA and CA for GP method (17).

### Accuracy of bone age assessment using GP method to predict actual age for children in Sabah

Our principal finding is that the GP method underestimates the forensic CA by 0.7 years in general. Specifically the GP method underestimates the forensic CA by 0.6 years in boys and 0.7 years in girls. In contrast, the GP method consistently overestimates the CA by about 0.5 to 1.1 years in indigenous Australian, Iranian, Turkish, Portuguese, Indian and undifferentiated Australian pediatric populations (2, 5–7). Consistent underestimation of CA by GP method in our study across all age groups as can be seen in the result section in Table 2 as well as boxplot Figure 3.

However, in some studies, it has been shown that in Asian children, particularly in boys, the GP method tends to underestimate CA, which is in agreement with our main findings. Kim et al. showed that the GP method underestimated CA in Korean boys (*n* = 135) by almost 0.5 years, while overestimated girls (*n* = 77) by 0.2 years (17). In another study, the GP method underestimated Asian boys from −0.35 to −1.23 years in the early to late childhood age group (*n* = 36) while overestimated girls by 0.14 to 0.33 years in girls of the same age group (*n* = 21) (4).

### Error metrics in describing accuracy of GP method in bone age assessment to forecast chronological age

In many of the published studies, the most commonly used accuracy metric, is what we label as accuracy measure 1, is the paired sample *t*-test to calculate any statistical significant difference between the two means of CA and BA in overall children and/or within age subgroups (4, 5, 18). The other common accuracy metrics that were used previously, is what we label as accuracy measure 2, was the mean absolute error, which is the overall mean of the difference between CA and BA for each cases which had been converted to absolute number (6, 7). This is important in order to gauge the performance of the GP method in order to correctly predict chronological age to a reasonable degree of accuracy, because implication of bone age estimation is important especially in medico-legal cases, follow up of children's health in pediatric clinics among others (2).

In our study we had presented both aspects of accuracy measures as described above, as depicted in Table 1. We have shown that in our population, using accuracy measure 1, that the GP method consistently underestimated chronological age with an estimated overall effect by 0.7 years. For accuracy measure 2, as described above, the statistical procedure performed were actually finding deviation of BA forecast from actual value of chronological age, which can be improved further by using the method more commonly known as Absolute Error.

To define an accuracy of a forecast instrument, several error metrics can be calculated such as mean absolute error (MAE), root of mean squared error (RMSE) and mean absolute percentage error (MAPE). Commonly used in business and marketing research when analyzing forecasting performance of an instrument, these error measurements metrics also have been commonly applied in deep learning and machine learning for computerized based development of forecast modeling.

### Performance comparison of error metrics with deep learning and machine learning for bone age assessment

In general, by consensus, a forecast or prediction model considered to have high accuracy when error metrics are low, thus, artificial intelligence (AI) technique such as machine learning (ML) and deep learning (DL) research have been using error metrics as described above to continuously and repeatedly measure several error metrics each time the AI tested several approach to predict or forecast an outcome.

Our study also utilizes mean absolute error, root of mean squared error and mean absolute percentage error to assess predictive performance of the GP method as an instrument to assess Bone Age to predict chronological age. In comparison to AI technique to predict chronological age using left hand radiograph, our study found that GP method done by radiologist outperform ML and DL technique performed by Wibisono et al. (MAE 0.7 vs. 1.23 years, MAPE 11.6% vs. 28.34%), undetermined to DL technique performed by Guo Longjun (MAE 0.7 vs. 0.56 years, RMSE 2.2 vs. 15.44), underperform to DL techniques based on GP method performed by Kim et al. (RMSE 2.2 vs. 0.42 and 0.7 years) (19–21).

### Prediction modeling for estimating chronological age using GP method

Based on our study, we found a strong correlation between bone age assessment by GP method and chronological age, which can be explained by a regression analysis as stated in the result. This can be a useful adjustment or modifier for bone age assessment in predicting chronological age. However, given the fact that there are a lot of potential confounders to explain the relationship between BA and CA, further research is needed in this area.

### Socioeconomic, nutritional status complex interplay in Sabahan children confounding underestimation of chronological age using GP method

The underestimation of BA in children in Sabah could be attributed to the inherently smaller physical size of our local children and also nutritional status of the child (22). Undernutrition is a challenge faced by as much as 34.7% of children under 5 years old in rural Sabah (22). The manifestation of undernutrition in Sabahan children belies a complex web of factors such as food security, basic sanitation, feeding practices, and socio-economic stability, all of which have direct impact on a young child's development. In a similar vein, adolescent girls may also be susceptible to socio-economic stressor leading to suboptimal health status (23). We postulate that the high BA standard deviations, errors could be contributed by many potential cofounders such as heterogeneous socio-economic landscape and unequal access to nutritional resources.

### Study limitation

There are several limitations to our study. There was no repeated measurement by the same radiologist after the initial assessment of left hand radiograph, thus inability to calculate intra-observer reliability was an issue. Other limitations include that we did not compute other potential confounders that could explain the consistent underestimation of chronological age by GP method such as nutritional and socio-economic status. Future research could focus on larger studies that take into account many other potential confounders to improve predictive performance of the GP method in Sabahan children to predict chronological age.

## Conclusion

Despite high interobserver reliability of GP Atlas estimation of BA to predict chronological age, it consistently, significantly underestimates the age of the child in all children, including both boys and girls, as well across all age groups albeit with an acceptably low amount of error metrics of measurement accuracy. Our findings suggest that locally validated GP Atlas or other type of assessments (artificial intelligence or machine learning) are needed for assessment of BA to accurately predict CA, since current GP Atlas standards significantly underestimated chronological age with minimal error for children in Sabah. A larger population-based study would be necessary for establishing a validated atlas of a bone age in Malaysia.

## Data Availability

The original contributions presented in the study are included in the article/supplementary material, further inquiries can be directed to the corresponding author/s.

## Key points

• Interobserver reliability was very high and strongly correlated for GP method to predict chronological age
• In Sabah, the GP Atlas significantly underestimated the chronological age with minimal error, of children between 9-18 years of age by 0.7 years. Subgroup analysis shown, GP Atlas underestimated chronological age by 0.6 years in boys and 0.7 years in girls.
• Underestimation of chronological age of children in Sabah using GP Atlas was associated with minimal errors. The mean absolute error (MAE) was 1.5 years, root of mean squared error (RMSE) was 2.2 years and mean absolute percentage error (MAPE) was 11.6%
• Underestimations of forensic age using the GP atlas were most significant at 13–13.9 years (−0.6 years), 17–17.9 years (−0.8 years) and 18.0–18.9 years (−1.5 years).

## References

[1] Greulich P Pyle S. Radiographic atlas of skeletal development of the hand and wrist. 2nd ed. Stanford, CA: Stanford University Press (1959).

[2] Franklin D Flavel A Noble J Swift L Karkhanis S. Forensic age estimation in living individuals: methodological considerations in the context of medico-legal practice. Res Rep Forensic Med Sci. (2015) 5:53–66. 10.2147/RRFMS.S75140

[3] Chaumoitre K Saliba-Serre B Adalian P Signoli M Leonetti G Panuel M. Forensic use of the greulich and pyle atlas: prediction intervals and relevance. Eur Radiol. (2017) 27(3):1032–43. 10.1007/s00330-016-4466-4 27357132

[4] Ontell FK Ivanovic M Ablin DS Barlow TW. Bone age in children of diverse ethnicity. Am J Roentgenol. (1996) 167(6):1395–8. 10.2214/ajr.167.6.8956565 8956565

[5] Büken B Şafak AA Yazıcı B Büken E Mayda AS. Is the assessment of bone age by the greulich–pyle method reliable at forensic age estimation for Turkish children? Forensic Sci Int. (2007) 173(2):146–53. 10.1016/j.forsciint.2007.02.023 17391883

[6] Patil ST Parchand MP Meshram MM Kamdi NY. Applicability of greulich and pyle skeletal age standards to Indian children. Forensic Sci Int. (2012) 216(1):200.e1–e4. 10.1016/j.forsciint.2011.09.022 22014975

[7] Moradi M Sirous M Morovatti P. The reliability of skeletal age determination in an Iranian sample using greulich and pyle method. Forensic Sci Int. (2012) 223(1):372.e1–e4. 10.1016/j.forsciint.2012.08.030 22986219

[8] U. N. H. C. for Refugees. “UNHCR Detention guidelines,” UNHCR. https://www.unhcr.org/publications/legal/505b10ee9/unhcr-detention-guidelines.html (accessed Mar. 12, 2023).

[9] Schmeling A Olze A Reisinger W Geserick G. Forensic age diagnostics of living people undergoing criminal proceedings. Forensic Sci Int. (2004) 144(2):243–5. 10.1016/j.forsciint.2004.04.059 15364396

[10] Albaker AB Aldhilan AS Alrabai HM AlHumaid S AlMogbil IH Alzaidy NFA Determination of bone age and its correlation to the chronological age based on the greulich and pyle method in Saudi Arabia. J Pharm Res Int. (2021) 33(60B):1186–95. 10.9734/jpri/2021/v33i60B34731

[11] Meyers LS Gamst GC Guarino AJ. Performing data analysis using IBM SPSS. Hoboken, NJ: John Wiley & Sons (2013).

[12] Breen AB Steen H Pripp A Gunderson R Sandberg Mentzoni HK Merckoll E A comparison of 3 different methods for assessment of skeletal age when treating leg-length discrepancies: an inter- and intra-observer study. Acta Orthop. (2022) 93:222–8. 10.2340/17453674.2021.1133 35019143

[13] Schober P Boer C Schwarte LA. Correlation coefficients: appropriate use and interpretation. Anesth Analg (2018) 126(5):1763–8. 10.1213/ANE.0000000000002864 29481436

[14] Koo TK Li MY. A guideline of selecting and reporting intraclass correlation coefficients for reliability research. J Chiropr Med. (2016) 15(2):155–63. 10.1016/j.jcm.2016.02.012 27330520 PMC4913118

[15] Pinchi V Skeletal age estimation for forensic purposes: a comparison of GP, TW2 and TW3 methods on an Italian sample. Forensic Sci Int. (2014) 238:83–90. 10.1016/j.forsciint.2014.02.030 24681971

[16] Berst MJ Dolan L Bogdanowicz MM Stevens MA Chow S Brandser EA. Effect of knowledge of chronologic age on the variability of pediatric bone age determined using the greulich and pyle standards. Am J Roentgenol. (2001) 176(2):507–10. 10.2214/ajr.176.2.1760507 11159105

[17] Kim JR Lee YS Yu J. Assessment of bone age in prepubertal healthy Korean children: comparison among the Korean standard bone age chart, greulich-pyle method, and tanner-whitehouse method. Korean J Radiol. (2015) 16(1):201. 10.3348/kjr.2015.16.1.201 25598691 PMC4296271

[18] Paxton ML Lamont AC Stillwell AP. The reliability of the greulich–pyle method in bone age determination among Australian children. J Med Imaging Radiat Oncol. (2013) 57(1):21–4. 10.1111/j.1754-9485.2012.02462.x 23374549

[19] Wibosono A Saputri M Mursanto P Rachmad J Alberto Wibowo A Deep learning and classic machine learning approach for automatic bone age assessment. 2019 4th Asia-Pacific Conference on Intelligent Robot Systems (ACIRS). (2019):235–40. 10.1109/ACIRS.2019.8935965

[20] Gao Y Zhu T Xu X. Bone age assessment based on deep convolution neural network incorporated with segmentation. Int J Comput Assist Radiol Surg. (2020) 15(12):1951–62. 10.1007/s11548-020-02266-0 32986142

[21] Kim JR Shim WH Yoon HM Hong SH Lee JS Cho YA Computerized bone age estimation using deep learning based program: evaluation of the accuracy and efficiency. Am J Roentgenol. (2017) 209(6):1374–80. 10.2214/AJR.17.18224 28898126

[22] How ETC Shahar S Robinson F Manah AM Ibrahim MY Jeffree MS Risk factors for undernutrition in children under five years of age in tenom, Sabah, Malaysia. Malays J Public Health Med. (2020) 20(1):71–81. 10.37268/mjphm/vol.20/no.1/art.557

[23] Azhary JMK Leng KL Razali N Sulaiman S Abd Wahab AV Adlan ASAA The prevalence of menstrual disorders and premenstrual syndrome among adolescent girls living in north Borneo, Malaysia: a questionnaire-based study. BMC Womens Health. (2022) 22(1):341. 10.1186/s12905-022-01929-1 35964024 PMC9375346

